# Supplementary figures and images for: Gait kinetics before and after total hip arthroplasty in people with unilateral hip osteoarthritis
Source: PLoS One. 2025 Jun 26;20(6):e0326502. doi: 10.1371/journal.pone.0326502 (PMC12200658; doi:10.1371/journal.pone.0326502)

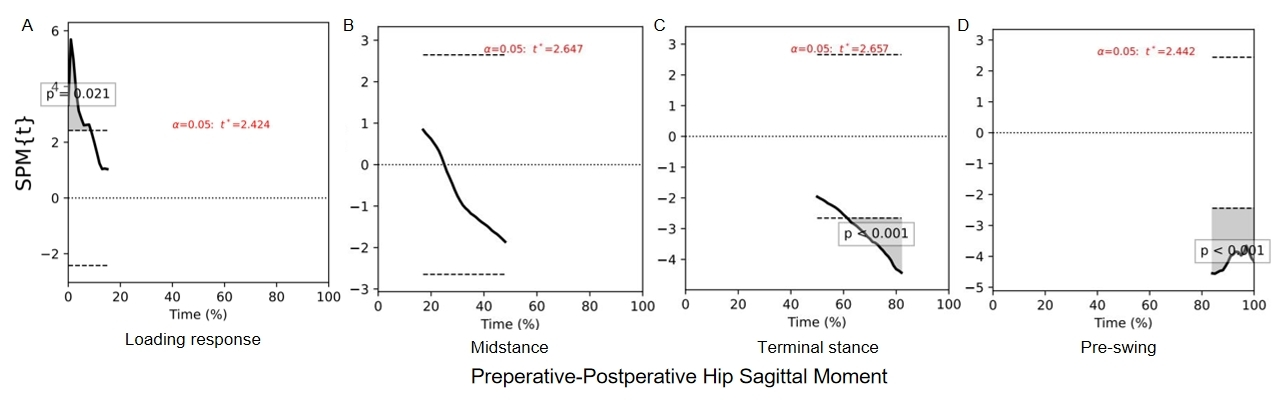

Supplement: S1 Fig — Loading response phase, (B) Mid-stance phase, (C)Terminal stance phase, (D) Pre-swing phase. The grey shaded areas indicate significant differences. (TIF) [file pone.0326502.s001.tif]

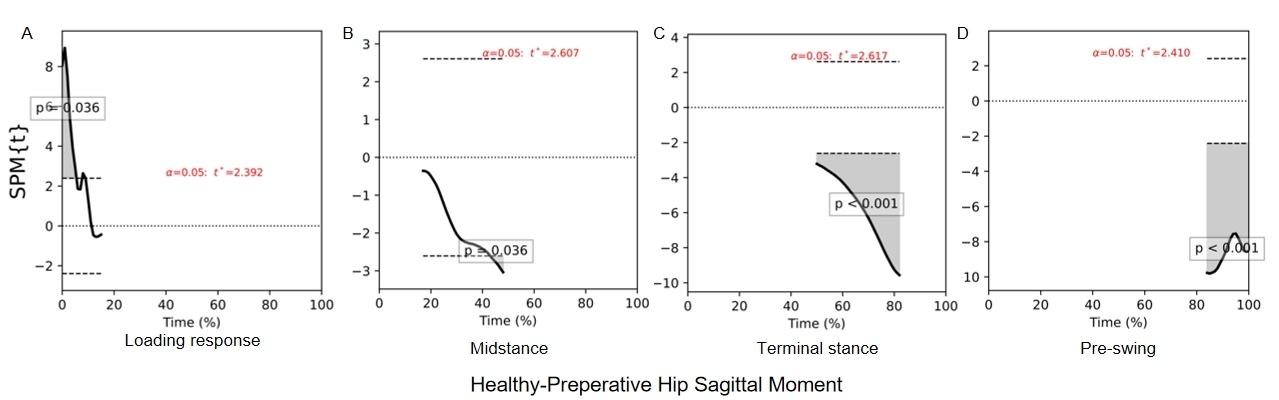

Supplement: S2 Fig — Loading response phase, (B) Mid-stance phase, (C)Terminal stance phase, (D) Pre-swing phase. The grey shaded areas indicate significant differences. (TIF) [file pone.0326502.s002.tif]

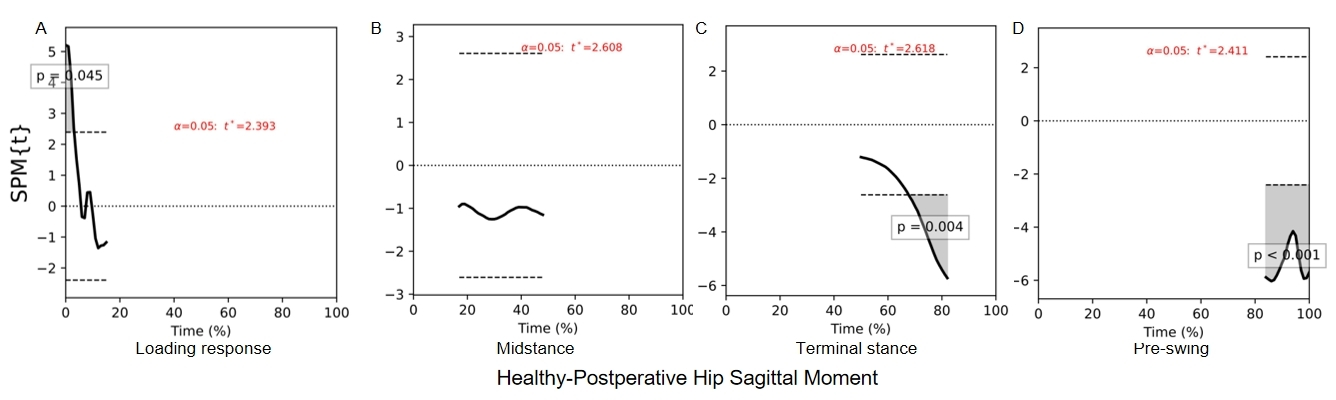

Supplement: S3 Fig — Loading response phase, (B) Mid-stance phase, (C)Terminal stance phase, (D) Pre-swing phase. The grey shaded areas indicate significant differences. (TIF) [file pone.0326502.s003.tif]

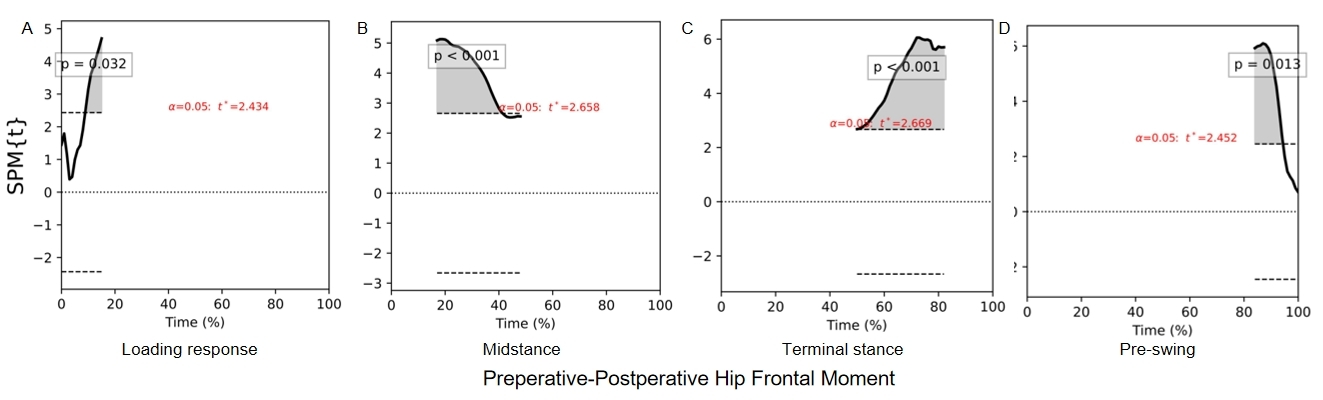

Supplement: S4 Fig — Loading response phase, (B) Mid-stance phase, (C)Terminal stance phase, (D) Pre-swing phase. The grey shaded areas indicate significant differences. (TIF) [file pone.0326502.s004.tif]

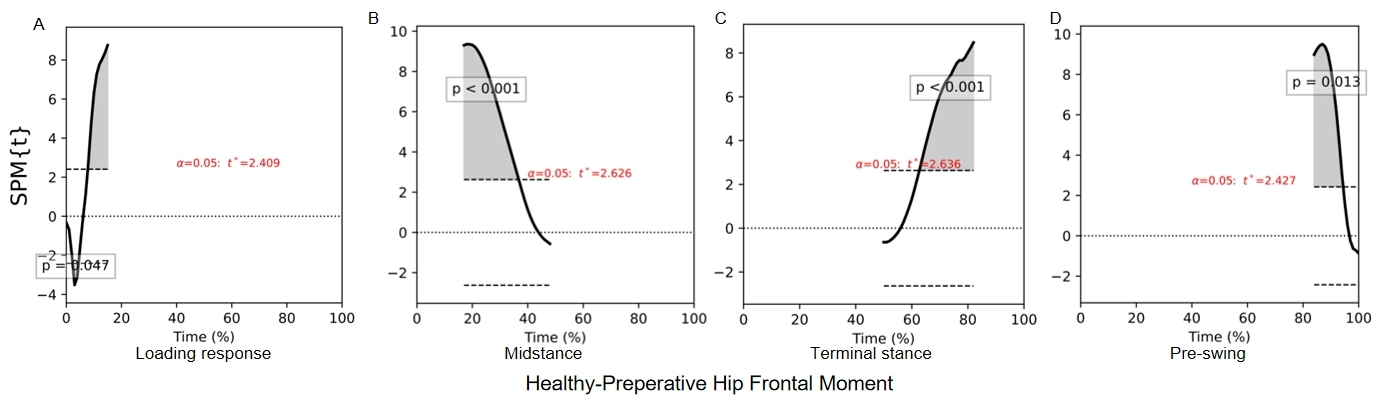

Supplement: S5 Fig — Loading response phase, (B) Mid-stance phase, (C)Terminal stance phase, (D) Pre-swing phase. The grey shaded areas indicate significant differences. (TIF) [file pone.0326502.s005.tif]

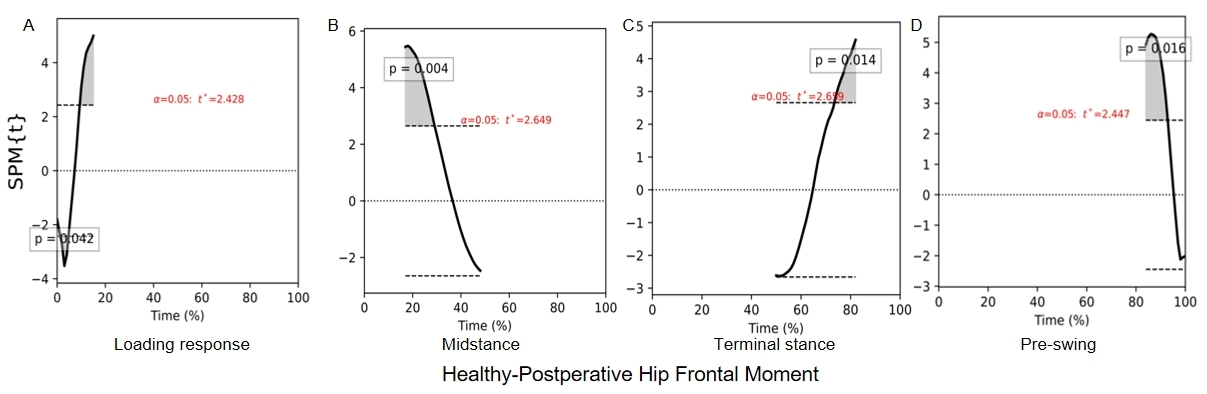

Supplement: S6 Fig — Loading response phase, (B) Mid-stance phase, (C)Terminal stance phase, (D) Pre-swing phase. The grey shaded areas indicate significant differences. (TIF) [file pone.0326502.s006.tif]

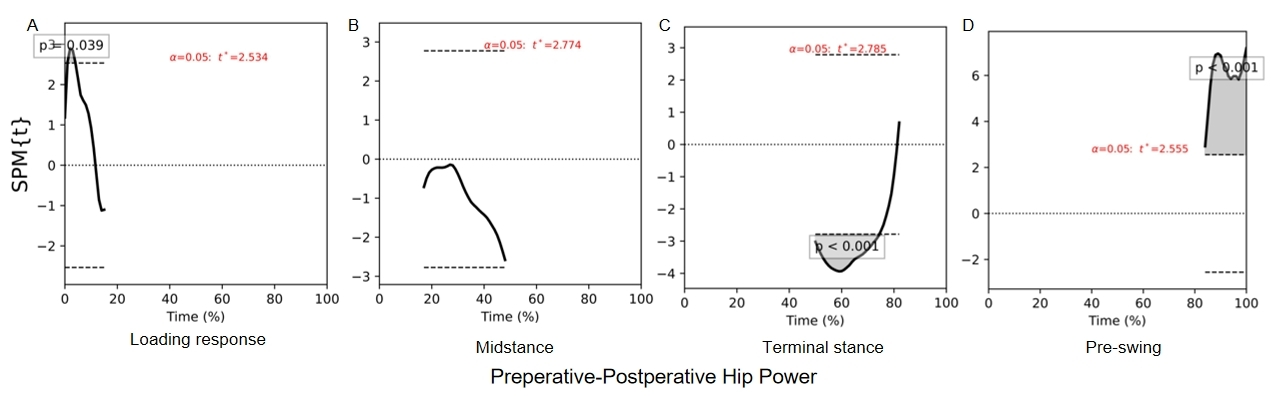

Supplement: S7 Fig — Loading response phase, (B) Mid-stance phase, (C)Terminal stance phase, (D) Pre-swing phase. The grey shaded areas indicate significant differences. (TIF) [file pone.0326502.s007.tif]

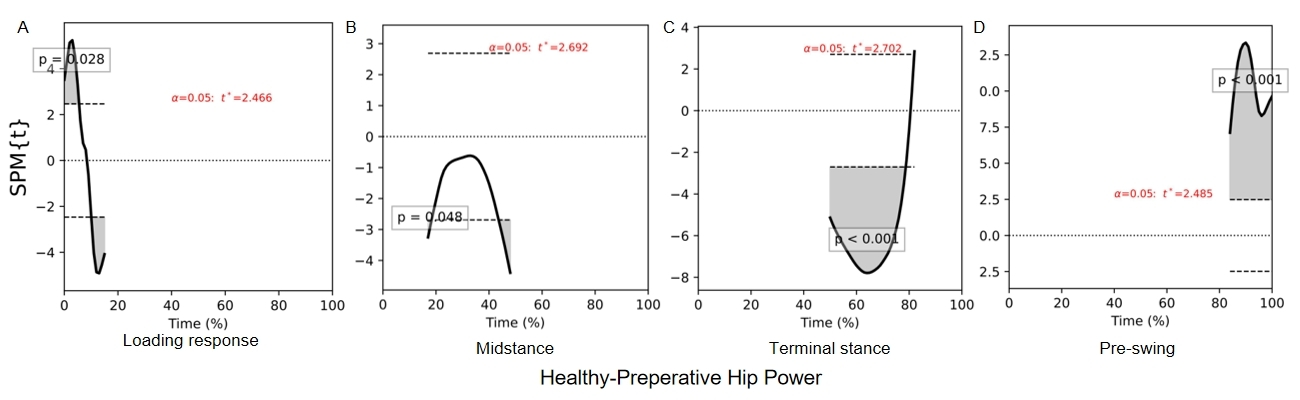

Supplement: S8 Fig — Loading response phase, (B) Mid-stance phase, (C)Terminal stance phase, (D) Pre-swing phase. The grey shaded areas indicate significant differences. (TIF) [file pone.0326502.s008.tif]

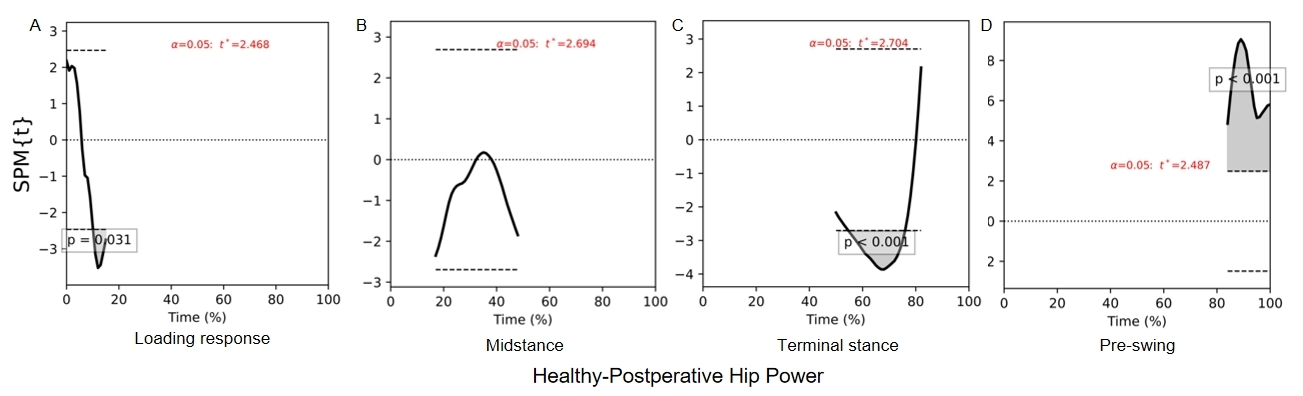

Supplement: S9 Fig — Loading response phase, (B) Mid-stance phase, (C)Terminal stance phase, (D) Pre-swing phase. The grey shaded areas indicate significant differences. (TIF) [file pone.0326502.s009.tif]
