# Supplementary material for: Gait kinetics before and after total hip arthroplasty in people with unilateral hip osteoarthritis
Source: PLoS One. 2025 Jun 26;20(6):e0326502. doi: 10.1371/journal.pone.0326502 (PMC12200658; doi:10.1371/journal.pone.0326502)
Supplement: S1 Table — (DOCX) [file pone.0326502.s010.docx]

**S1 Table. The multivariable regression results of hip sagittal moment.**

|  | **Group** | | **Age** | | **Sex** | | **BMI** | |
| --- | --- | --- | --- | --- | --- | --- | --- | --- |
|  | **t** | **P-value** | **t** | **P-value** | **t** | **P-value** | **t** | **P-value** |
| **Healthy VS Preoperative centroid1** | -8.094 | <0.001 | 1.035 | 0.303 | -1.724 | 0.087 | -0.069 | 0.945 |
| **Healthy VS Preoperative centroid2** | 6.315 | <0.001 | 2.429 | 0.016 | 2.214 | 0.029 | 1.985 | 0.049 |
| **Healthy VS Postoperative centroid1** | -4.451 | <0.001 | 1.106 | 0.271 | -1.577 | 0.117 | -1.061 | 0.290 |
| **Healthy VS Postoperative centroid2** | 3.398 | 0.001 | 3.734 | <0.001 | 1.449 | 0.150 | 2.308 | 0.023 |
