# Supplementary material for: Gait kinetics before and after total hip arthroplasty in people with unilateral hip osteoarthritis
Source: PLoS One. 2025 Jun 26;20(6):e0326502. doi: 10.1371/journal.pone.0326502 (PMC12200658; doi:10.1371/journal.pone.0326502)
Supplement: S2 Table — (DOCX) [file pone.0326502.s011.docx]

**S2 Table. The multivariable regression results of hip frontal moment.**

|  | **Group** | | **Age** | | **Sex** | | **BMI** | |
| --- | --- | --- | --- | --- | --- | --- | --- | --- |
|  | **t** | ***P*-value** | **t** | ***P*-value** | **t** | ***P*-value** | **t** | ***P*-value** |
| **Healthy VS Preoperative centroid1** | 2.442 | 0.016 | 2.126 | 0.035 | 4.219 | <0.001 | 0.737 | 0.462 |
| **Healthy VS Preoperative centroid2** | -8.196 | <0.001 | 1.634 | 0.105 | 0.803 | 0.423 | -0.674 | 0.501 |
| **Healthy VS Preoperative centroid3** | -7.061 | <0.001 | 0.608 | 0.544 | 0.151 | 0.880 | 0.067 | 0.947 |
| **Healthy VS Postoperative centroid1** | 3.031 | 0.003 | 2.336 | 0.021 | 4.361 | <0.001 | -0.691 | 0.491 |
| **Healthy VS Postoperative centroid2** | -4.899 | <0.001 | 1.328 | 0.187 | -0.358 | 0.721 | -0.597 | 0.552 |
| **Healthy VS Postoperative centroid3** | -4.005 | <0.001 | 0.046 | 0.964 | -0.023 | 0.981 | -0.053 | 0.958 |
