# Supplementary material for: Gait kinetics before and after total hip arthroplasty in people with unilateral hip osteoarthritis
Source: PLoS One. 2025 Jun 26;20(6):e0326502. doi: 10.1371/journal.pone.0326502 (PMC12200658; doi:10.1371/journal.pone.0326502)
Supplement: S3 Table — (DOCX) [file pone.0326502.s012.docx]

**S3 Table. The multivariable regression results of hip power.**

|  | **Group** | | **Age** | | **Sex** | | **BMI** | |
| --- | --- | --- | --- | --- | --- | --- | --- | --- |
|  | **t** | ***P*-value** | **t** | ***P*-value** | **t** | ***P*-value** | **t** | ***P*-value** |
| **Healthy VS Preoperative centroid1** | -4.838 | <0.001 | 0.436 | 0.663 | 0.624 | 0.534 | 0.838 | 0.404 |
| **Healthy VS Preoperative centroid2** | 2.920 | 0.004 | 4.129 | <0.001 | -1.737 | 0.085 | 0.466 | 0.642 |
| **Healthy VS Preoperative centroid3** | 5.558 | <0.001 | 2.061 | 0.041 | 2.253 | 0.026 | 1.739 | 0.084 |
| **Healthy VS Preoperative centroid4** | -11.193 | <0.001 | 1.502 | 0.135 | -0.703 | 0.484 | -1.588 | 0.115 |
| **Healthy VS Postoperative centroid1** | 2.084 | 0.039 | 4.141 | <0.001 | -1.026 | 0.307 | -0.600 | 0.550 |
| **Healthy VS Postoperative centroid2** | 2.623 | 0.010 | 2.766 | 0.006 | 1.012 | 0.313 | -0.255 | 0.799 |
| **Healthy VS Postoperative centroid3** | -6.524 | <0.001 | 0.029 | 0.977 | 0.022 | 0.983 | -2.063 | 0.041 |
