# Supplementary material for: Gait kinetics before and after total hip arthroplasty in people with unilateral hip osteoarthritis
Source: PLoS One. 2025 Jun 26;20(6):e0326502. doi: 10.1371/journal.pone.0326502 (PMC12200658; doi:10.1371/journal.pone.0326502)
Supplement: Attachment — Submitted filename: Response to Reviewers.docx [file pone.0326502.s013.docx]

Dear reviewers,

Thank you for your positive and constructive comments regarding our paper.

Here are our responses to your questions (The line numbers written in this file are the line numbers in the file *Revised Manuscript with Track Changes*):

Reviewer #1:

1.Please add "Sample size calculation in Method part".

**Response: We add sample size calculation in line 126-130: *The sample size was calculated using G*Power software version 3.1.9.7 (Universities, Düsseldorf, Germany). An effect size (d) of 0.81 was estimated based on data from Foucher et al. (26), which examined differences in hip extension motion before and after surgery. With a power of 80% and an alpha level of 0.05, the minimum sample size required to detect significant differences was determined to be 15.***

2. To calculate primary outcomes, confounding factors in dermographic data such as age, sex, BMI might effect the hip power and other kinametics. Multivariable regression with adjusting the confounders should be performed.

**Response: Thank you for your comment. We agree that demographic characteristics could have an important impact on the reported results. Since SPM1D currently does not support the analysis of individual predictors in a multiple regression model, we conducted a traditional multivariable regression analysis to explore the potential effects of these variables. Across all conditions, group membership remained a significant predictor. The results of the multivariable regression have been included in Supplementary Table 1-3, and the corresponding statistical analysis has been added to the Methods section (lines 163–174).**

Reviewer #2:

1. Title

- please change your title related to your study

- include pre-post operative THA, unilateral OA and gait kinetics

**Response: Thank you for the suggestion, We changed the title into “Gait Kinetics Before and After Total Hip Arthroplasty in Unilateral Osteoarthritis”.**

Abstract

2.- background: include the problem statement in this part

**Response:** **The following problem statement was added in Line 21-22: ‘‘*Limited studies have examined changes in hip kinetics throughout the entire stance phase.’***

3.- method: include how you reconstruct into 3D in one sentence

**Response: We added this sentence in line 28-29: “*Motion capture data obtained using the Plug-in Gait marker set was analyzed and modeled in Visual3D*”**

4.- careful with conclusion of walking pattern gradually normalize while you're only study for gait kinetic

**Response: We changed *“walking pattern”* into *“hip kinetics”* in line 46.**

Introduction

5.- please add more information about walking speed (Line 84). joint moment and power (Line 85), as this is critical to your study

**Response: We enhance the description about the walking speed and gait kinetic in line 90-97. “*Furthermore, it is well-established that greater joint moments and power are strongly associated with faster gait speeds, reflecting more efficient and dynamic locomotion (19, 20). However, previous research has shown that individuals often experience a significant decline in walking speed that persists for over a year following surgery (21). This finding is consistent with other studies reporting long-term gait speed deficits after THA (16, 22, 23). Given the critical role of gait speed in functional recovery and its relationship with joint kinetics, this study also examined the effects of walking speed on hip kinetics in individuals post-THA.* ”**

Method

6.- please include 1 figure showing your work on gait kinetic using Visual3D with markers related.

**Response: We have added Fig. 1** **to illustrate the data processing workflow for gait kinetics using Visual3D.**

7.- please divide into 4 segments of stance phase (initial contact, loading response, mid stance and terminal stance) while describing your data. this will be your main contribution of this study because all data already been given by Bertaux et al. (2022).

**Response: Thank you for your comment. We have divided the stance phase into four subphases for detailed analysis. The changes in each subphase were analyzed and described accordingly (line 191-195; 223-226; line 245-247). Additionally, separate SPM1D analyses were conducted for each subphase (Line 150-154, with the results presented in Supplementary Figures 1–9. And the subphase that showed significant differences was indicated in Table 4-6.**

Result

8.- figure: include the legend within the figure (ease reader to comprehend)

**Response: Thank you for your suggestion. We added the legend into figures.**

9.- the subtopic is mixed, example in sagittal hip moment included frontal and hip power. similar to another subtopic.

**Response: Thank you for your suggestion. We have reformated the tables, separating hip sagittal moment, hip frontal moment, and hip power into 3 tables to make the subtopics clearer.**

10.- please aligned with your objective, easier to comprehend and demonstrated that you achieved it or not.

**Response: Thank you for the comment, we have revised the objective statement and revised to align with the results sessions. Line 99-106: *“The objectives of this study were threefold: (1) to identify differences in hip moment between participants who have unilateral hip OA after 6 months of THA, (2) to compare them with healthy controls, and (3) to determine the impact of walking speed on hip moment in participants undergoing THA. The same research question was studied in hip power. We hypothesised that the hip moment and power of participants who have unilateral hip OA would increase by 6 months after THA. We also hypothesised that no difference would be identifiable between participants who underwent THA and healthy individuals.”***

Discussion

11.- as suggested in result section, discussed based on your objective/hypothesis, easier for readers to comprehend.

**Response: Thank you for your suggestion. We revised the objective in lines 99-106 so that it aligns with the flow of discussion.**

12.- include limitation on the last paragraph in this section

**Response: Thank you for your suggestion. The limitation is now the last paragraph in this section.**

References

13.- if possible, include more recent publications (5 years prior)

**Response: We have included several latest publications to ensure our literature review is up to date.**

***Martinez L, Noé N, Beldame J, Matsoukis J, Poirier T, Brunel H, et al. Quantitative gait analysis after total hip arthroplasty through a minimally invasive direct anterior approach: A case control study. Orthopaedics & Traumatology: Surgery & Research. 2022;108(6):103214.***

***Lalevée M, Martinez L, Rey B, Beldame J, Matsoukis J, Poirier T, et al. Gait analysis after total hip arthroplasty by direct minimally invasive anterolateral approach: A controlled study. Orthopaedics & Traumatology: Surgery & Research. 2023;109(7):103521.***

***Macie, A., Matson, T., & Schinkel-Ivy, A. (2023). Age affects the relationships between kinematics and postural stability during gait. Gait & Posture, 102, 86-92.***

***Rowe, E., Beauchamp, M. K., & Wilson, J. A. (2021). Age and sex differences in normative gait patterns. Gait & posture, 88, 109-115.***
