# Supplementary material for: Gait kinetics before and after total hip arthroplasty in people with unilateral hip osteoarthritis
Source: PLoS One. 2025 Jun 26;20(6):e0326502. doi: 10.1371/journal.pone.0326502 (PMC12200658; doi:10.1371/journal.pone.0326502)
Supplement: Attachment — Submitted filename: Response_to_Reviewers_auresp_2.docx [file pone.0326502.s014.docx]

Dear reviewers,

Thank you for your positive and constructive comments regarding our paper.

Here are our responses to your questions (The line numbers written in this file are the line numbers in the file *Revised Manuscript with Track Changes*):

Reviewer #3: Review comments

1. Although hip arthroplasty (THA) in the treatment of hip osteoarthritis (OA) is introduced, the current research background and existing literature are not reviewed in detail. For example, how do you specifically define the difference between "full recovery" and "partial recovery"? Specific research findings on the different stages of postoperative recovery (e.g., 6 months) are less mentioned, which could be further fleshed out. It is recommended to add background information on the recovery process at different time points after hip replacement, especially at 6 months, as well as a comparison with a healthy population, to help readers better understand the complexity of postoperative patient recovery.

**Response: Thank you for your suggestion. The literature gap you pointed out is precisely the important innovation point of this research. Through a literature search, we found that most of the existing studies after THA focus on short-term (3 weeks /6 weeks) or long-term (≥1 year) follow-up, and there are few studies lasting 6 months. To highlight this research gap, we have added relevant paragraphs in line 92-96: “Six months after THA represents a clinically important time-point when most patients have achieved formal gait rehabilitation programs and achieved substantial functional improvement (7, 20). Horstmann et al. found that although patients showed improvements in gait symmetry and joint range of motion by six months, their dynamic hip and knee motions remain significantly lower than those of healthy controls, and muscle activity patterns differ substantially (4). While Casartelli et al. demonstrated that at six months post-THA, patients walk with velocity, cadence, and support times that are comparable to healthy individuals (21).”**

**We have also added relevant paragraphs in line 65-72: In other words, even after surgery, patients who undergo THA still exhibit significant differences in gait patterns compared to the normal population (9). Studies have shown that impaired postoperative walking function can lead to fatigue, subsequently limiting patients' ability to perform daily activities and reducing their quality of life (10). Therefore, it is essential to prioritize gait assessment as a key functional activity following THA. Quantitative gait analysis is required to better understand gait mechanics, as these mechanisms may underlie the functional limitations observed in patients who have undergone the procedure (11).**

2. Although many studies have explored changes in gait dynamics, there are still deficiencies in gait pattern recognition and gait differences among different sports groups (such as high-low mileage runners) in the current literature. Some new methods have been proposed to realize the recognition of human gait patterns, which not only provide a new perspective for sports science, but also provide inspiration for clinical gait analysis. for example, A new method proposed for realizing human gait pattern recognition: A new method proposed for realizing human gait pattern recognition: Inspirations for the application of sports and clinical gait analysis (https://doi.org/10.1016/j.gaitpost.2023.10.019). In addition, another study used machine learning to explain differences in gait patterns between high-mileage and low-mileage runners, Explaining the differences of gait patterns between high and low mileage runners with machine. Explaining the differences of gait patterns between high and low mileage runners with machine Learning (https://doi.org/10.1038/s41598-022-07054-1). However, most of the existing literature focuses on athlete groups or specific gait pattern recognition techniques, and there has been no systematic discussion on the changes of gait dynamics in patients with hip replacement (THA) before and after surgery, especially the specific differences in different gait substages."

**Response: Thank you for your suggestion. We added this paragraph in lines 108-121, “Although many studies have explored changes in gait dynamics, there are still deficiencies in gait pattern recognition and gait differences among different sports groups (such as high-low mileage runners) in the current literature (28, 29). Some new methods have been proposed to realise the recognition of human gait patterns, which not only provide a new perspective for sports science but also provide inspiration for clinical gait analysis. For instance, Xu and Zhou (28) utilised Deep Neural Networks (DNN) and Layer-wise Relevance Propagation (LRP) to analyse gait pattern differences between high- and low-mileage runners, identifying ankle and knee kinematics and kinetics in the sagittal and transverse planes as key discriminators. In addition, another study used machine learning to explain differences in gait patterns between high-mileage and low-mileage runners (29). However, most of the existing literature focuses on athlete groups or specific gait pattern recognition techniques, and there has been no systematic discussion on the changes of gait dynamics in patients with hip replacement (THA) before and after surgery, especially the specific differences in different subphases of the stance phase.”**

***Reference added***

***A new method proposed for realizing human gait pattern recognition: Inspirations for the application of sports and clinical gait analysis (https://doi.org/10.1016/j.gaitpost.2023.10.019). Explaining the differences of gait patterns between high and low mileage runners with machine Learning (https://doi.org/10.1038/s41598-022-07054-1).***

3. Although the study divided gait into four sub-stages for analysis, the specific physiological significance of these stages and the segmentation criteria were not elaborated in the method section. Please clarify the physiological significance of each sub-stage of gait and the reasons for choosing these stages, to help readers understand the importance of each stage for gait recovery.

**Response: Thank you for your suggestion. We added the specific physiological significance of four subphases and the segmentation criteria in the methods in line 173-183: “ The loading response was defined that one side of foot initially strikes on the ground until the contralateral foot leaves from the ground and normalized to 16 data points, serving as the critical transition period for weight acceptance and shock absorption. The mid-stance phase was the single-leg support period from contralateral toe-off to when the body's center of mass aligns over the stance foot and normalized to 33 data points, representing the body's challenge to maintain stability on one limb. The terminal stance was the period from heel rise to contralateral heel contact and normalized to 34 data points, representing the body's final push-off mechanism. The pre-swing phase was defined that both feet on the ground until one side of foot leaves the ground and normalized to 18 data points, serving as the critical transition from stance to swing (11, 34, 35). ”**

***Reference added***

***Zhao Y, Raza W, Arnold G, Li P, Wang W. A Preliminary Study on Kinetic Analysis of Ground Reaction Force and Impulse During Gait in Patients With Total Hip Replacement and Implication for Rehabilitation. Orthop Surg. 2024;16(12):3162-78.***

***Fukaya T, Mutsuzaki H, Nakano W, Mori K. Smoothness of the knee joint movement during the stance phase in patients with severe knee osteoarthritis. Asia-Pacific Journal of Sports Medicine, Arthroscopy, Rehabilitation and Technology. 2018;14:1-5.***

***Alijanpour E, Russell DM. Gait phase normalization resolves the problem of different phases being compared in gait cycle normalization. J Biomech. 2024;173:112253.***

4. Although the changes in the gait of patients after hip replacement were discussed, some factors (such as muscle strength and pain) that did not fully recover were not discussed in depth. It is recommended to further explore the role of muscle strength, pain and other physiological factors during postoperative recovery to explain why these factors may lead to incomplete recovery of gait and hip dynamics.

**Response: Thank you for your suggestion. We explained more about the incomplete recovery of gait and hip dynamics in line 337-343: “Therefore, despite these postoperative improvements, full recovery of gait and joint kinetics may be limited by persistent muscle strength deficits. For instance, Ismailidis et al. found that even 24 months after surgery, hip abductor torque ratios had not returned to normal levels in many patients, suggesting that residual weakness may hinder complete restoration of frontal plane stability (49). Moreover, Friesenbichler et al. reported that while maximal muscle strength improved after THA, explosive strength remained impaired at 6 months, especially in hip flexors, which are critical for swing-phase initiation (50).”**

***Reference***

***Ismailidis P, Kvarda P, Vach W, Cadosch D, Appenzeller-Herzog C, Mündermann A. Abductor Muscle Strength Deficit in Patients After Total Hip Arthroplasty: A Systematic Review and Meta-Analysis. J Arthroplasty. 2021;36(8):3015-27.***

***Friesenbichler B, Casartelli NC, Wellauer V, Item-Glatthorn JF, Ferguson SJ, Leunig M, et al. Explosive and maximal strength before and 6 months after total hip arthroplasty. J Orthop Res. 2018;36(1):425-31.***

Reviewer #4: This study suffers from several critical issues that affect its scientific validity and credibility. First, despite the use of a publicly available dataset, the source, characterization, and quality control details of the dataset were severely lacking, and the completeness and accuracy of the data were not verified. Second, the sample selection and exclusion criteria were unclear, with insufficient explanation of the rationale for sample sizes exceeding the required values, and the lack of transparent exclusion criteria may have led to selection bias. For data processing, although Visual3D was used for the analysis, details of parameter settings and validation were not provided, the choice of low-pass filter (50 Hz) may not be applicable to all gait characteristics, and the accuracy of the gait event detection method was not validated or analyzed for sensitivity. In the statistical analysis, the authors did not perform a valid correction for multiple comparisons, the assumed conditions and applicability of the SPM1D model were not adequately validated, and the multivariate regression analysis lacked detailed description and ignored covariate interaction effects. Finally, the interpretation of the study results is too superficial, lacks mechanistic analysis, and has limited scientific contribution. In view of the above issues, this study is not eligible for publication in its current form, and the authors are advised to resubmit it with significant revisions in terms of data transparency, methodological rigor, and interpretation of results.

**Response: We appreciate the reviewer’s comment regarding the dataset used in our study. However, we respectfully disagree with the assertion that the source, characterisation, and quality control of the dataset were lacking. The dataset used in this study is from Bertaux et al., “Gait analysis dataset of healthy volunteers and patients before and 6 months after total hip arthroplasty,” published in Scientific Data (2022, Nature Portfolio) [DOI: 10.1038/s41597-022-01113-5]. This is a peer-reviewed, FAIR-compliant dataset explicitly designed for clinical gait analysis research. It is hosted by a trusted institutional repository and curated following high standards of data transparency and reproducibility. The data has passed the quality review of the platform itself before being used and has been cleaned and screened again in our research.**

**Second, we thank the reviewer for raising concerns regarding the sample selection process and potential selection bias. However, we respectfully clarify that our manuscript does address these issues and provides a transparent account of the exclusion criteria. Table 2 summarises the reasons for participant data exclusion.**

**For data processing, Visual3D (C-Motion, Inc.) was used for the full kinematic and kinetic processing pipeline, including model creation, filtering, joint moment computation, and gait event detection. We used a standard lower-limb model with six degrees of freedom, and joint centers were computed based on anthropometric estimations. Marker trajectories and ground reaction forces were filtered using a 4th-order zero-lag Butterworth filter. The filter cut-off frequency is discussed below. These settings are consistent with best practices in gait analysis and with prior work using the same dataset. The choice of a 50 Hz cut-off was based on the high fidelity of the motion capture system (acquisition at 200 Hz) and force plates (1,000 Hz). Previous work has shown that higher cut-off frequencies can preserve subtle gait events and fast transitions, particularly in studies involving high-resolution signals or derivative calculations (e.g., joint powers). Importantly, we applied the same filter across all trials and subjects, maintaining internal consistency.**

**For the statistical analysis, we confirm that all statistical parametric mapping (SPM1D) analyses employed random field theory (RFT)-based inference, which provides family-wise error correction appropriate for smooth, time-continuous biomechanical data. In accordance with SPM1D conventions, a cluster-based thresholding approach was applied at α = 0.05. Clusters were only considered statistically significant if the supra-threshold area exceeded the RFT-derived critical threshold and a corresponding cluster-wise p-value was reported, as detailed in the manuscript.We thank the reviewer for pointing out the need for further explanation of the multivariable regression. As stated in the manuscript, SPM1D does not currently support multiple regression involving more than one independent variable. Therefore, to explore the influence of demographic covariates such as age, sex, and BMI, we extracted scalar values at significant SPM1D cluster centroids and conducted multivariate linear regression using SPSS. These models included group membership as a fixed factor and demographic variables as covariates. While interaction terms were not included initially, we have now reanalyzed the data by including relevant two-way interactions (e.g., group × sex, group × BMI), and the results remain consistent: group remained a significant predictor in all models. Additional details on model structure, regression diagnostics, and interaction effect evaluation have been added to the Statistical Analysis section and Supplementary Tables S1–S3.**

**We carefully interpreted our results in relation to previously published findings and biomechanical theories of post-THA recovery. Our discussion addresses both the temporal-spatial and kinetic changes following THA and offers explanations grounded in muscle strength recovery and compensatory movement strategies. We link our kinetic findings (e.g., persistent asymmetries in hip moments and powers) to underlying physiological constraints, including incomplete recovery of hip abductor strength and residual pain.**
